# Delayed Diagnosis of Tricuspid Atresia: A Case Report

**DOI:** 10.31729/jnma.8969

**Published:** 2025-05-31

**Authors:** Ramita Nath Yogi, Som Raj Awasthi, Arun Kumar Mandal, Kiran Shahi, Kamal Prasad Thani, Sidhartha Pradhan

**Affiliations:** 1 Health Service Office Kalikot Manma, Kalikot, Nepal; 2 District Hospital Darcula, Darchula, Nepal; 3 Kanti Children's Hospital, Maharajgung, Kathmandu, Nepal; 4 Department of Pediatrics, Karnali Academy of Health Sciences, Chandannath, Jumla, Nepal; 5 Department of Cardiac Surgery, Shahid Gangalal National Health Centre, Bansbari, Kathmandu, Nepal

**Keywords:** *case report*, *echocardiography*, *surgery*, *tricuspid atresia*

## Abstract

Tricuspid atresia is a rare cyanotic congenital heart disease that impairs oxygenation. A 16-month-old female child from a rural area in Nepal presented with shortness of breath, cyanosis of the lips and extremities, feeding difficulties, and severe acute malnutrition. The patient had been diagnosed with cyanotic congenital heart disease at nine months but had sought treatment from shamans due to financial constraints. Diagnostic echocardiography at the referred center revealed tricuspid atresia type IB with a ventricular septal defect. While awaiting surgery, the child collapsed and was revived after cardiopulmonary resuscitation. Emergency surgery was then performed. After surgery, the child had a stormy recovery and was subsequently discharged home in stable condition.

## INTRODUCTION

Tricuspid atresia is a rare cyanotic congenital heart disease (CHD), with a prevalence of about 0.5 per 10000 live births.^1^ It involves the congenital agenesis or absent of the tricuspid valve, preventing blood flow from the right atrium to the right ventricle, thence to pulmonary circulation, impairing oxygenation.^2^ Here we present a case of a sixteen months old female child from rural Nepal who presented with, shortness of breath, cyanosis of the lips and extremities, feeding difficulties, and severe acute malnutrition and was later diagnosed as tricupid atresia. This case aims to highlight the crucial role of early diagnosis of tricuspid atresia and its management.

## CASE REPORT

A sixteen months' female child was brought to zonal hospital located at rural Nepal, with shortness of breath, bluish discoloration of the lips and peripheries since a year. Initially, the symptoms were present while crying or feeding, but had become progressive and was present at rest since the last three days, thus, the present visit to the hospital.

The baby was born at term at the local health post, which was also the place for scheduled ante-natal checkups, the mother followed. The documents surrounding the event of the birth are missing, but the mother states that anamoly scan was not done and the perinatal period was uneventful. The child apparently had delayed milestones on interrogating the parents. The child had been seen at a local zonal hospital for ear discharge at nine months of age. During the checkup, apart from the specific treatment, the parents had been advised to take the child to a higher center for cardiac evaluation. This advice was ignored by the parents, and had instead sought the help of traditional healers.

Upon examination at sixteen months of age, the child was ill looking, with severe malnutrition and cyanosis. The respiratory rate was 36 breaths per minute and SpO2 was 78% in room air. On chest auscultation, a systolic murmur, best heard at left lateral sternal border at third and fourth intercostal spaces was evident. Echocardiography revealed complex congenital heart disease with suspicion of tricuspid atresia. The child was referred to tertiary cardiac center for further management.

At the tertiary referral centre, echocardiography revealed abdominal situs solitus, levocardia, tricuspid atresia type IB with atrial septal defect (ASD), ventricular septal defect (VSD) with left ventricular ejection fraction 60%. Hemoglobin was 11.5 mg/dl. Rests of the parameter were within normal range.

**Figure 1:**
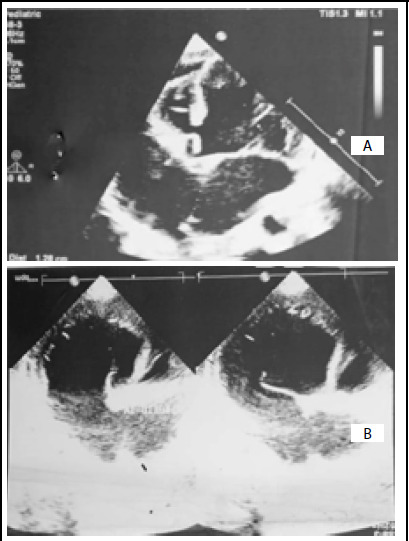
Echocardiography of congenital tricuspid atresia type IB with ASD and VSD. (A); Apical four chamber view. Atretic tricuspid valve, hypoplastic RV, ASD and normal left chambers. (B); Apical four chamber view. Atretic tricuspid valve with normal functioning mitral valve.

**Figure 2:**
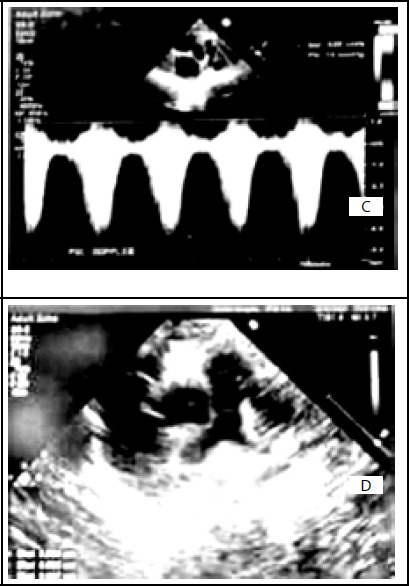
Echocardiography of congenital tricuspid atresia type IB with ASD and VSD. (C); Doppler study of pulmonary valve. Increased gradient across the stenotic pulmonary valve. (D); Parasternal short axis view. The pulmonary artery segment from the valve till the bifurcation.

On the next day of admission patient collapsed and revived after cardiopulmonary resuscitation (CPR) and intubated. Oxygen saturation did not improve even after intubation and mechanical ventilation; hence, emergency bidirectional cavo-pulmonary shunt and excision of interatrial septum (IAS) was done. The operative findings revealed right superior vena cava (SVC) draining into dilated right atrium (RA), hypoplastic right ventricle (RV), small main pulmonary artery (PA), right PA and left PA were confluent without narrowing, small ductus arteriosus present, tricuspid orifice was absent and replaced by fibrous membrane, well-formed septum primum with a large ostium secundum ASD and VSD. During surgery, right SVC dissected from its origin to its termination, azygos vein was divided between two hemo-clips, SVC divided from its right atrial end and the atrium closed with two layers of 6-0 poly-propylene suture. Right PA was dissected and SVC anastomosed to right PA in end to side fashion with 7-0 polypropylene suture. The heart was then arrested using modified St. Thomas II Cardioplegia solution. Right atriotomy and excision of IAS remnant was done.

The child was extubated on second post-operative day but had to be re-intubated again the same day as oxygen saturation could not be maintained. The child was extubated 24 hours later and put on bubble continuous positive airway pressure (CPAP) support due to respiratory issues. The child stayed in ICU for 13 days and required inotropic and inodilator support in the immediate post-operative period. The PA pressure was optimized with milrinone then changed to sildenafil and bosentan. Other medications prescribed were enalapril, spironolactone and furosemide. She had two episodes of fever with no focus of infection and was managed with paracetamol. Patient was discharged after 19 days of hospital stay with the above medications as well as cefixime and flucloxacillin for five days for haziness on chest x-ray detected prior to discharge. At time of discharge SpO2 was 94% at room air. The rest of the examination was within normal limits. Patient was doing continuous visit at zonal hospital, thereafter at every 2 months for medication. Patient condition including feeding and developmental milestone were improving. Fontan procedure was advised when child reached at the age of 5-7 years.

## DISCUSSION

A systematic review and meta-analysis on CHD done in Nepal showed prevalence of critical CHD including tricuspid atresia among cyanotic CHD was 12.6 % but the prevalence of tricuspid atresia was not specified. The age of onset of symptoms was not studied. In this context, age of onset of symptoms which was 4 months, though this may vary according to the severity of pulmonary stenosis.^3^

Among 779 cases of cyanotic CHD treated over a five years' period at Shahid Gangalal National Heart Centre, the most common age range for presentation of cyanotic heart disease was between one to five years. Another survey was conducted in Eastern Nepal at BPKIHS, Dharan, reported the mean age of diagnosis of CHD was 22.31 ± 34.08 months. These are strong evidences which suggest a delay in diagnosis of CHD in Nepal.^4,5^

In a case report from India, tricuspid atresia with PDA was prenatally diagnosed using fetal echocardiography. Another case report with review of literature reported a case diagnosed within an hour of birth. Both cases received early management leading to improved quality of life. In the present case, due to lack of fetal echocardiogram, ante-natal diagnosis was missed. The child's heart condition was only diagnosed during a hospital visit for another condition at 9 months of age.^6,7^

A retrospective cohort study has shown varying rates of prenatal detection of Cyanotic CHD; from 13% in Slovak Republic to 87% in France -Rhone. This indicates the significance of policies, scanning protocol and technical expertise in early diagnosis and management which coincide with the need of this case in rural area.^8^

Though this child was managed successfully, the treatment course was stormy and life was at significant risk. This is supported by a review article that found early diagnosis and management of tricuspid atresia significantly reduce morbidity and mortality.^9^

Despite the diagnosis at nine months, the family first visited local shamans due to financial constraints and lack of awareness regarding the disease and treatment modalities. They sought medical management only after the child's condition worsened. A multilevel analysis on economic burden and cost determinants of CHD done in rural southwest China found that higher level of education lead to better use of health resources. Most of the CHD were managed with surgery, long term medication and regular follow up leading to financial burden in low socioeconomic family alike this child's, which also determine disease outcome.^10^

Early diagnosis can provide adequate time for management plan, reduce patient suffering and improve outcome. Health facilities in rural area should have trained clinicians and better equipment for anomaly scans and CHD screening during antenatal and neonatal period. There should be a robust community health delivery system, where infants with poor milestones and weight gain are referred to a district level health center, which scans for anomalies and refers to relevant referral center, in this case, the national heart center.
